# Sensitive Scalp: A Possible Association With the Use of Hair Conditioners

**DOI:** 10.3389/fmed.2020.596544

**Published:** 2021-03-15

**Authors:** Emilie Brenaut, Laurent Misery, Cécile Legeas, Alain-Claude Roudot, Anne-Sophie Ficheux

**Affiliations:** ^1^Department of Dermatology, CHU Brest, Brest, France; ^2^University of Brest, LIEN, Brest, France

**Keywords:** sensitive scalp, sensitive skin, cosmetic products, hair products, 3S questionnaire, BoSS questionnaire, sensitive scale

## Abstract

A sensitive scalp is defined by the occurrence of unpleasant sensations (tingling, burning, pain, pruritus) triggered by stimuli that should not cause such sensations. Environmental factors, particularly cosmetics, can be triggering factors. The aims of this study were to assess hair cosmetic product consumption in subjects with sensitive scalp and to perform a clinical evaluation of sensitive scalp. After a dermatological examination, women between the ages of 18 and 65 years with or without a sensitive scalp completed different questionnaires. Their use of hair cosmetics (frequency, amount per application) was recorded. A total of 160 women with a mean age of 41 years were included. Twenty-seven subjects presented with seborrheic dermatitis or psoriasis, so only 133 were included in the analysis. Five percent of the subjects declared they had a very sensitive scalp, 25% had a sensitive scalp, 38% had a slightly sensitive scalp, and 32% had a scalp that was not sensitive. The mean sensitive scalp score (3S) score was 3.7 ± 1.6 in the very sensitive scalp group, 3.6 ± 2.1 in the sensitive group, 1.2 ± 1.2 in the slightly sensitive group and 0.1 ± 0.4 in the non-sensitive group. Two groups were analyzed: the 56 subjects with a sensitive scalp (3S ≥ 2 score) and the 56 subjects with a null 3S score. In the sensitive scalp group, 89% suffered from itch, and 45% suffered from tingling. No parameter (hormonal status, smoking, age, phototype, BMI) was associated with the 3S score. No differences in the exposure to shampoos and masks between the two groups were noted. The exposure to hair conditioners was significantly higher in the sensitive scalp group than in the group without sensitive scalp. Itch is the main symptom of a sensitive scalp. The frequency of a sensitive scalp was lower than that previously described when the subjects with scalp dermatosis were excluded. The amount of hair conditioners used was significantly higher in subjects with sensitive scalp than in those without sensitive scalp, suggesting a possible link.

## Introduction

Sensitive skin has been defined by the sensitive-skin special interest group in the International Forum for the Study of Itch (IFSI) as the occurrence of unpleasant sensations (stinging, burning, pain, pruritus, and tingling sensations) in response to stimuli that normally should not provoke such sensations (1). These symptoms are not explained by any other skin disease. Sensitive skin affects approximately half of the population and is more frequent in women than in men (2, 3). The pathophysiological mechanisms have been debated, and several hypotheses exist (4). Sensitive skin can be considered a result of a decrease in the skin tolerance threshold, which thereby impairs barrier function and leads to abnormalities in the cutaneous nervous system, making the skin hyperreactive (4–6). Sensitive skin can therefore be the clinical expression of neurogenic inflammation and can be modulated by many factors (7). Triggering factors of sensitive skin can be physical (ultraviolet, heat, cold, and wind), chemical (cosmetics, water, and pollutants), and occasionally psychological (stress) (8). A recent meta-analysis showed that the most frequent triggering factors were cosmetics, with an odds ratio of >7 (8). However, no specific information on the actual consumption of cosmetic products among people with sensitive skin is available. Thus, the SENSICOS study was designed to assess the relationship between cosmetic use and sensitive skin.

A sensitive scalp is one of the most frequent complaints related to sensitive skin. Sensitive skin can be located on different areas of the body, and sensitive scalp is a specific condition because of the presence of hairs on the head, it is associated with different symptoms (9) and nerves innervate the scalp in a specific pattern (10, 11). The first study on this condition showed that 44% of French people, more commonly women, suffer from sensitive scalp (9). Environmental factors, particularly cosmetics, probably trigger sensitive scalp (12). The aim of this study was to evaluate hair cosmetic product consumption in subjects with sensitive scalp and to clinically evaluate the factors associated with, the symptoms of, and the correlations among scores related to sensitive scalp.

## Materials and Methods

This monocentric prospective study took place in the dermatology department of the University Hospital of Brest. The aim of the SENSICOS study was to compare the cosmetic product consumption of subjects presenting with sensitive skin in a group of adult women. Recruitment was carried out in three ways: with an e-mail sent to the employees of the hospital, with an announcement in a free local newspaper and by contacting our acquaintances. In the announcement, it was written that women between 18 and 65 years old with or without sensitive skin and with any level of cosmetic product consumption were eligible.

The aim of this study was to evaluate hair cosmetic product consumption in subjects with sensitive scalp and to clinically evaluate parameters associated with sensitive scalp (associated factors, symptoms, correlations with different scores).

The inclusion criteria were women between 18 and 65 years old without any skin diseases on the face. The exclusion criteria were as follows: males, women younger than 18 years or older than 65 years, individuals with a skin facial disease (such as eczema, rosacea, acne, psoriasis, etc.), and individuals who refuse to participate. A total of 160 subjects needed to be included in this study.

The subjects were invited to visit our dermatology department for an appointment with a dermatologist. An information letter was given to all patients. The subjects did not receive any compensation. A clinical examination was performed to verify the absence of a skin disease to ensure the subject met the inclusion and exclusion criteria.

Then, all subjects completed questionnaires concerning the following factors:

- Sociodemographic and clinical data, including age, weight, hormonal status, smoking status, and socioprofessional category
- Sensitive skin factors, which were evaluated by the following question about sensitive skin, “Is your skin very sensitive, sensitive, slightly sensitive, or not sensitive at all?” Moreover, the volunteers were asked about the frequency at which they experience sensitive skin. Then, the sensitive scale, which includes items on 10 signs felt on the face in the last 3 days (skin irritability, tingling, burning and warmth, tightness, itching, pain, general discomfort and flushes) was used. The scores ranged from 0 (no intensity) to 10 (unbearable intensity). The final score (SS-10) varied between 0 and 100 (13).
- Sensitive scalp factors, which were assessed by the following question about sensitive scalp, “Is your scalp very sensitive, sensitive, slightly sensitive, not sensitive at all?” Moreover, the volunteers were asked about the frequency at which they experience sensitive scalp. The sensitive scalp score (3S) was used: it includes items (which symptoms have you experienced on your scalp?) on five sensations (itching, tingling, tightness, pain and burning sensations), with scores ranging from 0 (absent) to 4 (unbearable), so the total score ranged between 0 and 20 (14).
- The burden of sensitive skin, which was assessed with the BoSS questionnaire (15). It consists of 14 questions, which are scored from 0 (never) to 4 (always) and concern three dimensions: personal care, daily life and appearance. The total score ranges from 0 to 56.
- Cosmetic product consumption for scalp, which was assessed by the frequency of use of shampoos, hair conditioners, masks, hair dyes, and hair bleach. The subjects brought all the cosmetic products they were using at least once a week for the scalp. Then, they dispensed their shampoos, hair conditioners and masks in their hand and applied them to their scalp, and the weight of their bottles before and after application were noted to determine the amount of product used per application. The composition of each product was noted. The exposure to each product (mg/kg bw/day) was calculated with the following formula: [frequency (day^−1^) x amount (mg/use)]/body weight (kg bw).

Statistical analysis was performed using XLSTAT 2019.1 (Addinsoft, Paris, France). Descriptive statistics are presented as the means and standard deviations (SDs) for the quantitative variables and as percentages for the qualitative variables. For group comparisons, we used the Mann-Whitney test, the Kruskal-Wallis test or Chi^2^ test, as appropriate. A correlation analysis (Pearson's correlation coefficient) was used to assess the link between the 5 items involved in the calculation of the 3S score and the final score and the correlations between the following 3 scores: 3S, SS-10 and BoSS. *p* < 0.05 indicated statistical significance.

The study was registered on ClinicalTrials.gov with the title “Impact of Exposure to Cosmetics on Sensitive Skin (SENSICOS),” and the identifier was NCT03958968. The study protocol was approved by the ethics committee of Brest (29BRC18.0078).

## Results

### Recruitment and Subjects

One hundred and sixty women volunteers were included between July and October 2019. Concerning recruitment, 38% of the subjects were acquaintances, 36% responded to the announcement in the local newspaper, and 26% responded to the e-mail sent to the hospital employees. The average age of the participants was 41 ±13 years (19–65). Among the 160 subjects included in the SENSICOS study, 27 presented with a skin disease of the scalp (24 had seborrheic dermatitis, and 3 had psoriasis). The 133 subjects analyzed had a normal skin on the scalp, without redness. The mean 3S score was 1.6 ± 1.9 in subjects without scalp dermatosis (*n* = 133) and 3.3 ± 3.2 in subjects with seborrheic dermatitis (*n* = 24), and this difference was significant (*p* = 0.017). Among the 160 subjects, 11% of the participants reported having a “very sensitive scalp,” 48% reported having a “sensitive scalp,” 30% reported having a “slightly sensitive scalp,” and 11% reported having a scalp that was “not sensitive at all.” Participants with psoriasis or seborrheic dermatitis of the scalp were excluded from the rest of the analysis because the definition of sensitive skin excluded the presence of a skin disease. Consequently, the data from only 133 subjects were analyzed.

### Presence of Sensitive Scalp

**Figure 1:**
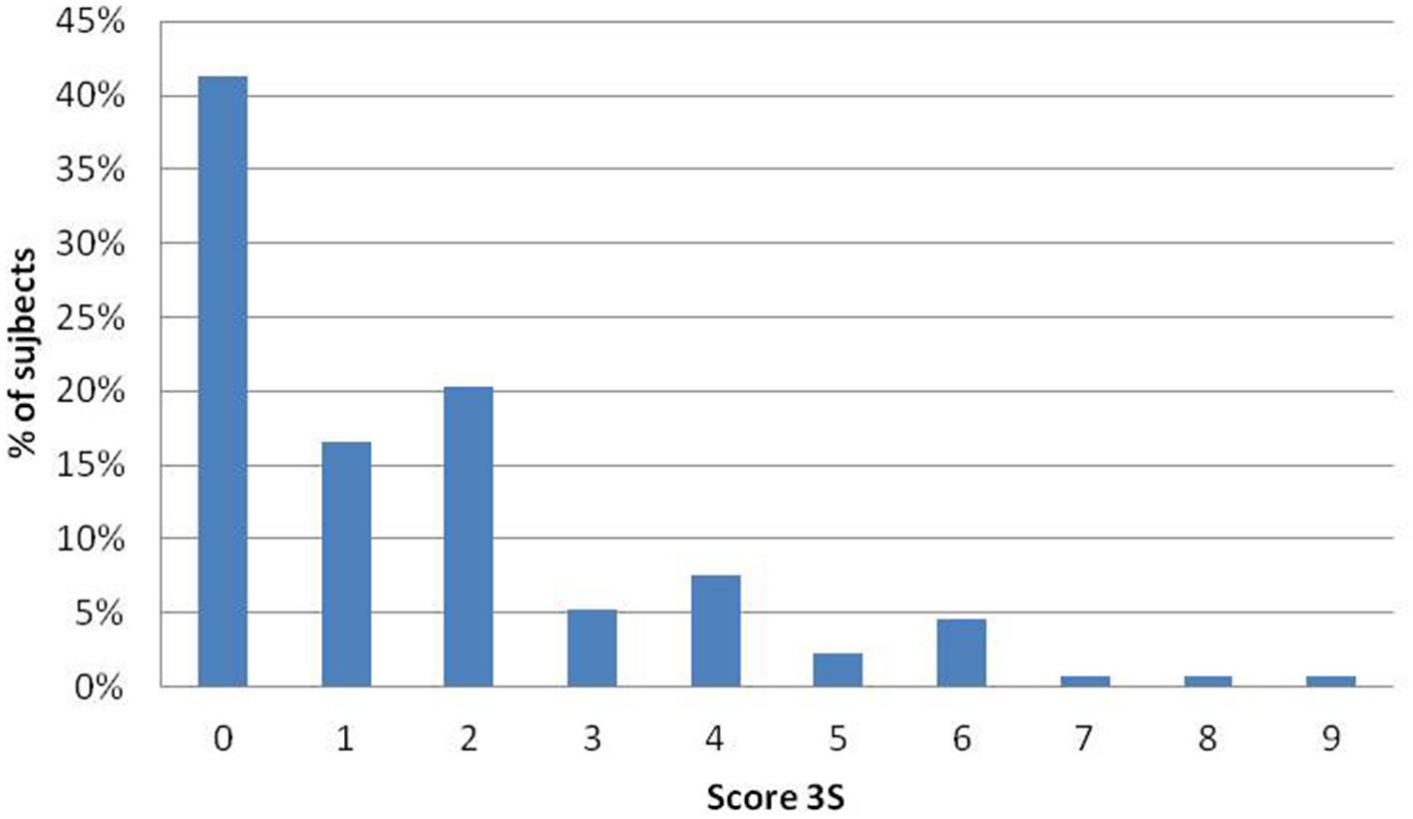
Distribution of the 3S scores in the group of 133 subjects without scalp dermatosis.

**Figure 2:**
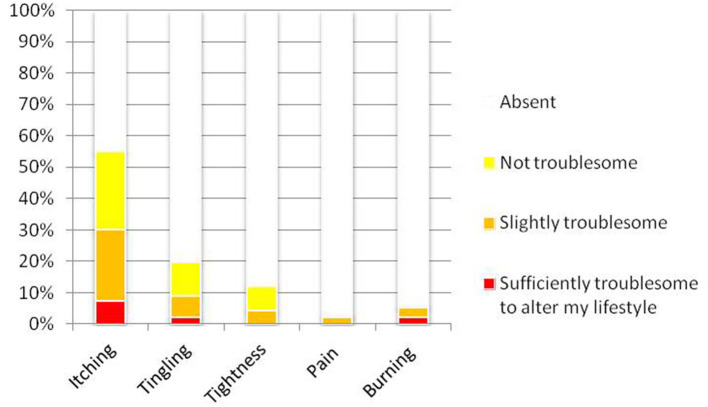
Intensity of each item of the 3S questionnaire.

**Figure 3:**
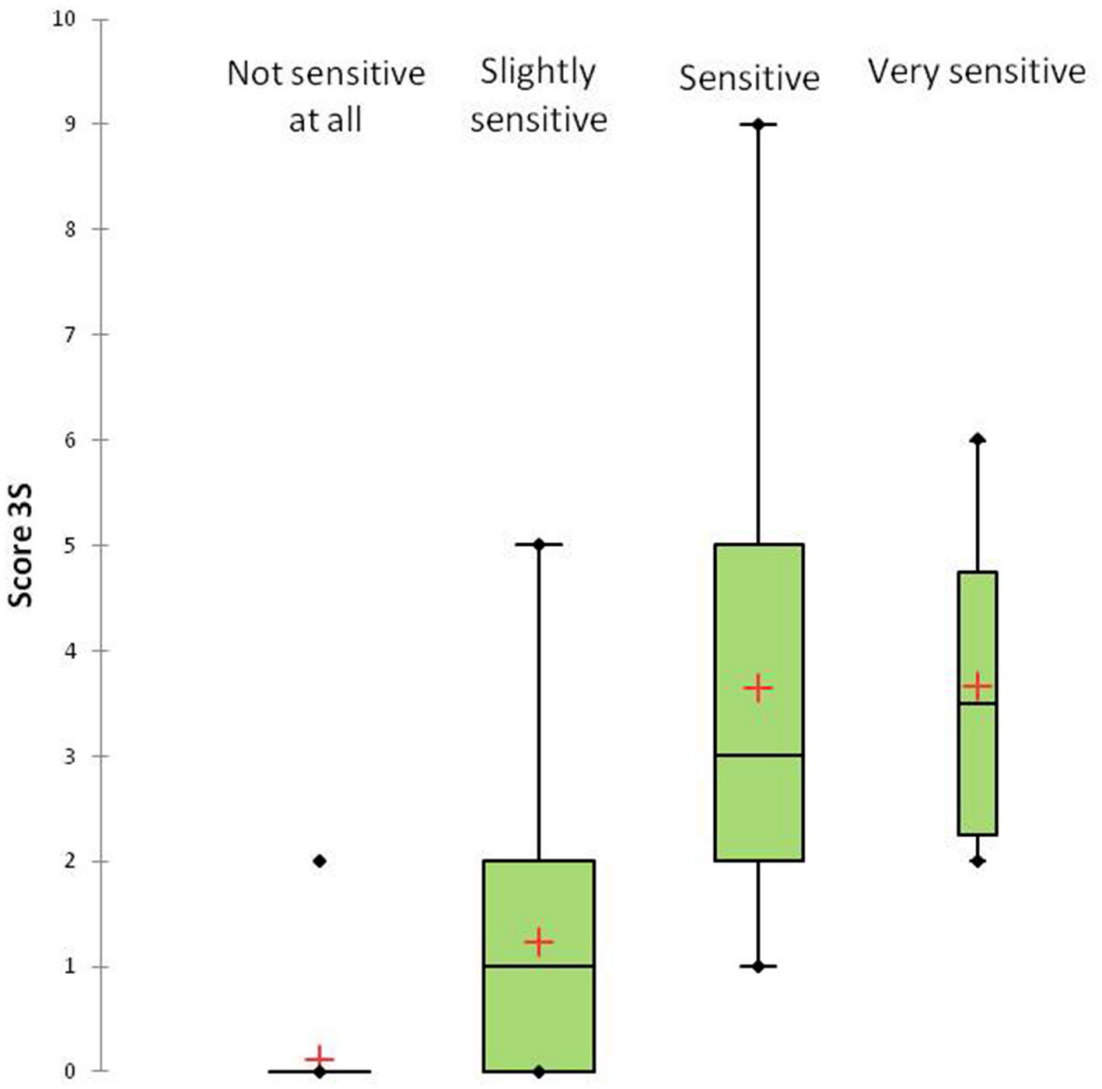
3S scores stratified by the severity of sensitive scalp.

Five percent of the participants reported having a “very sensitive scalp,” 25% reported having a “sensitive scalp,” 38% reported having a “slightly sensitive scalp,” and 32% reported having a scalp that was “not sensitive at all.” The mean 3S score was 1.6 ± 1.9 (range 0–9). The maximum 3S score possible is 20. The distribution of the 3S scores is presented in Figure 1 (range 0 to 9). The intensity of each item of the 3S questionnaire is presented in Figure 2. The mean 3S score was 0.1 ± 0.4 in the “not sensitive at all” group, 1.2 ± 1.2 in the “slightly sensitive scalp” group, 3.6 ± 2.1 in the “sensitive scalp” group and 3.7 ± 1.6 in the “very sensitive scalp” group (Figure 3). The Kruskal-Wallis statistical test was used to compare the 4 groups. Statistically significant differences were observed among the 4 groups (*p* < 0.0001).

### Items of the 3S Questionnaire

**Figure 4:**
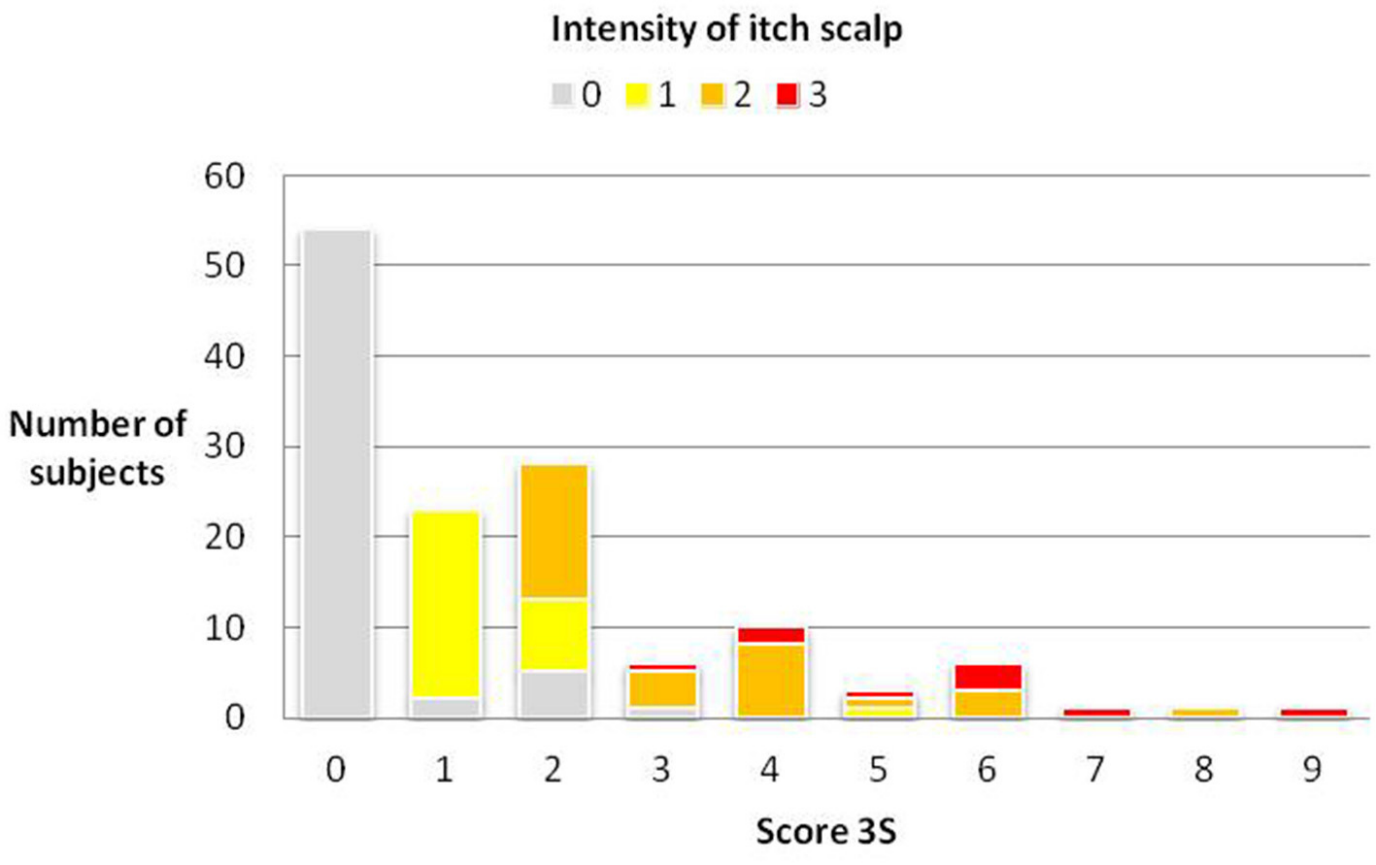
Distribution of subjects according to the intensity of the itch scalp and the 3S score.

A positive and statistically significant correlation was found between each of the 5 items involved in the calculation of the 3S score and the final score. The strongest correlation was with itching (0.832), followed by tingling (0.666), tightness (0.555), burning (0.532), and pain (0.264). The distribution of subjects according to the intensity of the itch scalp and the 3S score is presented in Figure 4.

### Factors Associated With a Sensitive Scalp

None of the general medical or dermatological parameters (menopause, smoking, phototype, type of skin, erythema, age, weight, height) were correlated with the 3S score (Pearson's correlation).

### Correlation Between the Three Scores: The 3S, SS-10, and BoSS Scores

**Figure 5:**
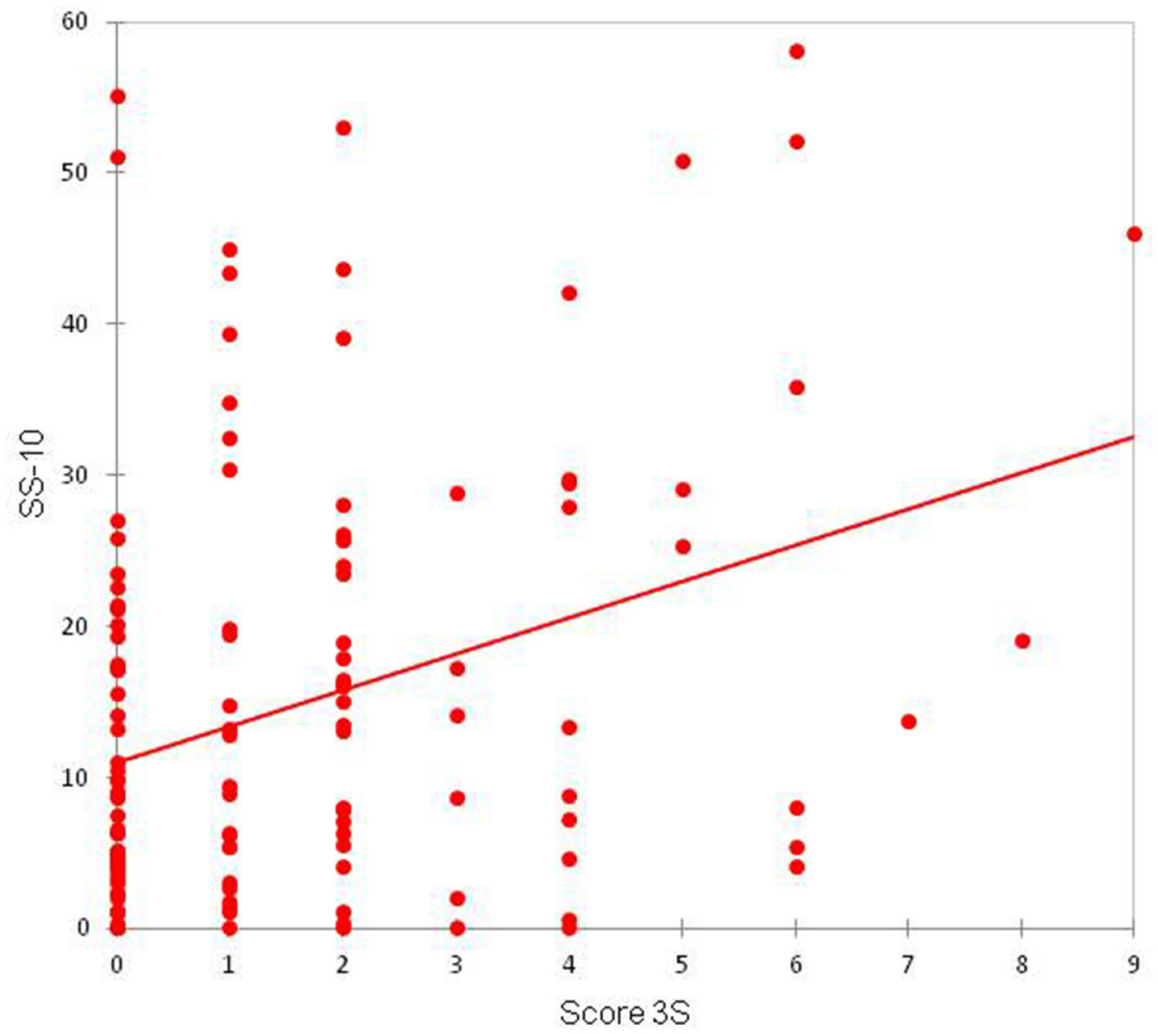
Correlation between the SS-10 and 3S scores.

**Figure 6:**
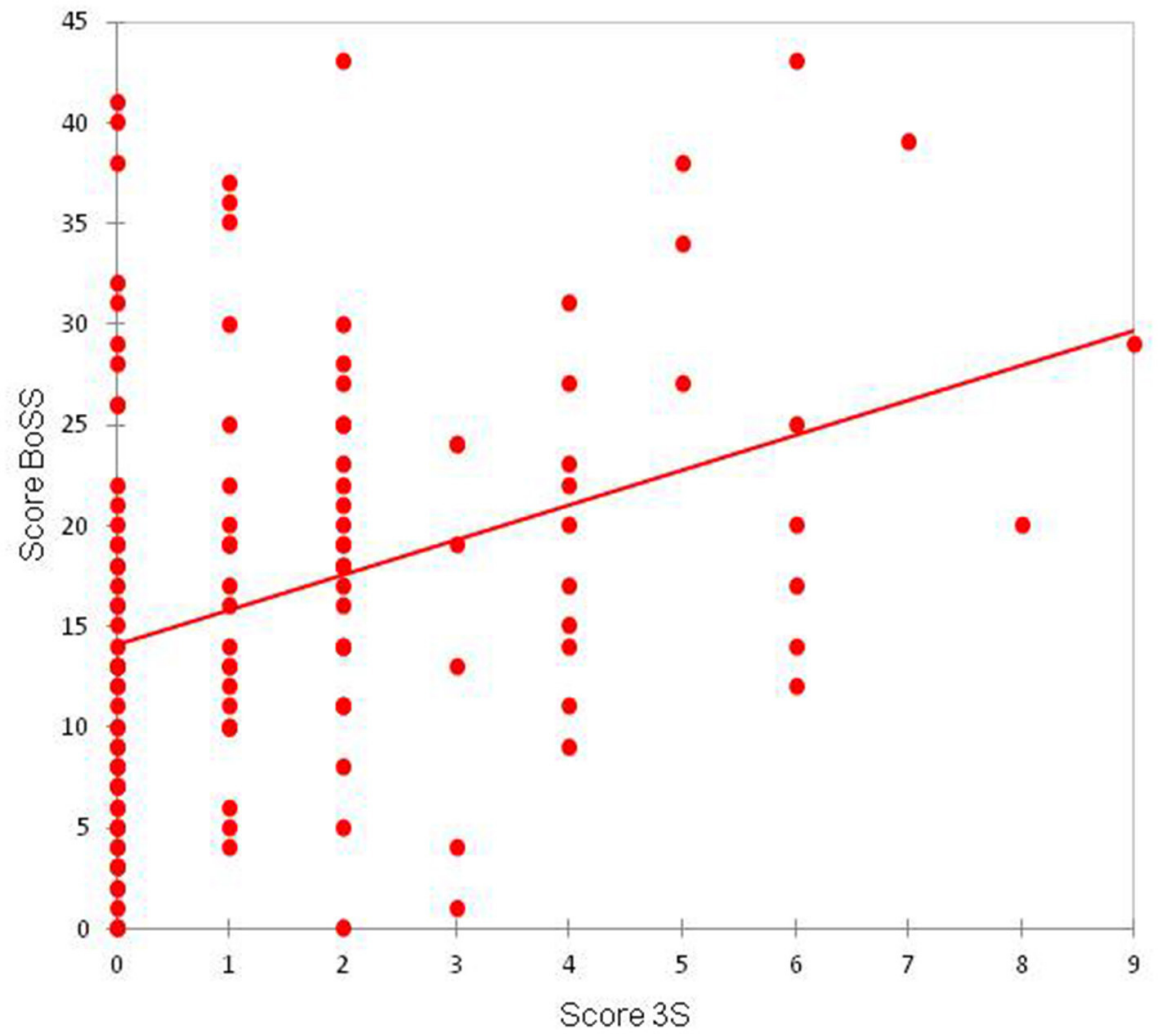
Correlation between the 3S and BoSS scores.

A positive (0.329) and statistically significant correlation (*p* < 0.01) was observed between the SS-10 score and the 3S score (Figure 5). A positive (0.328) and statistically significant correlation (*p* < 0.01) was observed between the BoSS score and the 3S score (Figure 6).

### Comparisons of the Groups With and Without a Sensitive Scalp

Two groups were formed according to the 3S score: the non-sensitive scalp group included the 55 subjects with a 3S score equal to 0, and the sensitive scalp group included the 55 subjects with a 3S score higher than or equal to 2. In the sensitive scalp group, 90.9% of the subjects presented with itching, 45.5% presented with tingling, 27.3% presented with tightness, 14.5% presented with burning, and 7.3% presented with pain.

### Cosmetic Product Consumption

Shampoos were used by 100% of the subjects in the two groups. Hair conditioners were used by 40% of the women in the non-sensitive scalp group vs. 53.6% in the sensitive scalp group (*p* = 0.15). Masks were used by 40% of the women in the non-sensitive scalp group vs. 48.2% in the sensitive scalp group (*p* = 0.38). Hair dyes were used by 41% of the women in the non-sensitive scalp group vs. 52.7% in the sensitive scalp group (*p* = 0.18). Hair bleach was used by 23.6% of the women in the non-sensitive scalp group vs. 20% of the persons in the sensitive scalp group (*p* = 0.64). The frequency, amount and exposure to shampoos, hair conditioners and masks in the 2 groups are presented in Table 1. There were no data concerning the amount or exposure to hair dyes and hair bleach because the subjects brought all the cosmetic products that they used at least once a week for the scalp. A statistically significant difference was observed in the amount of hair conditioner applied per use: this amount was lower in the non-sensitive scalp group than in the sensitive scalp group (*p* = 0.03). No statistically significant difference was observed between these two groups in the other parameters that were studied.

**Table 1:** The frequency of use, amount used and exposure to hair cosmetic products in the two groups.

		**Sensitive scalp group (*n =* 56)**	**Non sensitive scalp group** ** (*n =* 56)**	
Shampoos	Percentage of users	100%	100%	*p =* 1
	Frequency (day^−1^)	0.56 ± 0.37 (*n =* 52)	0.55 ± 0.38 (*n =* 52)	*p =* 0.81
	Amount (g/use)	6.40 ± 5.39 (*n =* 53)	4.90 ± 2.91 (*n =* 48)	*p =* 0.45
	Exposition (mg/kg bw/day)	60.54 ± 68.91 (n = 50)	45.35 ± 36.33 (*n =* 47)	*p =* 0.71
Hair conditioners	Percentage of users	53.6%	40%	*p =* 0.15
	Frequency (day^−1^)	0.32 ± 0.27 (*n =* 28)	0.37 ± 0.30 (*n =* 20)	*p =* 0.48
	Amount (g/use)	5.48 ± 5.87 (*n =* 19)	2.88 ± 3.75 (*n =* 11)	*p =* 0.03
	Exposition (mg/kg bw/day)	28.52 ± 25.20 (*n =* 18)	18.16 ± 25.12 (*n =* 11)	*p =* 0.07
Masks	Percentage of users	48.2%	40%	*p =* 0.38
	Frequency (day^−1^)	0.11 ± 0.09 (*n =* 27)	0.13 ± 00.9 (*n =* 21)	*p =* 0.73
	Amount (g/use)	6.19 ± 4.03 (*n =* 15)	5.38 ± 4.28 (*n =* 10)	*p =* 0.68
	Exposition (mg/kg bw/day)	0.014 ± 0.0155 (*n =* 15)	0.014 ± 0.014 (*n =* 10)	p =0.93
Hair dyes	Percentage of users	52.7%	41%	*p =* 0.18
	Frequency (year^−1^)	6.43 ± 2.62 (*n =* 23)	6.66 ± 3.10 (*n =* 12)	*P =* 0.92
Hair bleach	Percentage of users	20%	23.6%	*p =* 0.64
	Frequency (year^−1^)	5.71 ± 3.14 (*n =* 7)	5.71 ± 2.14 (*n =* 7)	Not evaluated

### Types of Cosmetic Products

Shampoos for sensitive scalp were used by 20% of the women in the sensitive scalp group and 12.7% of the women in the non-sensitive scalp group (*p* = 0.3). Organic shampoos were used by 25.4% of the women in the sensitive scalp group and 20% of the women in the non-sensitive scalp group (*p* = 0.49). Shampoos for children were used by 12.7% of the women in the sensitive scalp group and 5.4% of the women in the non-sensitive scalp group (*p* = 0.18).

Hair care products for sensitive scalp were used by 12.7% of the women in the sensitive scalp group and 7.2% of the women in the non-sensitive scalp group (*p* = 0.3). Organic hair care products were used by 20% of the women in the sensitive scalp group and 7.2% of the women in the non-sensitive scalp group (*p* = 0.05).

### Point of Purchase

Concerning the point of purchase for shampoos, 63.6, 29.1, 10.9, 9.1, 9.1, and 5.8% of the subjects with sensitive scalp and 63.1, 20, 14.5, 9.1, 5.8, and 3.8% of the subjects with non-sensitive scalp said they buy their products from supermarkets, pharmacies, beauty brands, the internet, hair salons, and organic supermarkets, respectively (*p* = 0.2, *p* = 0.2, *p* = 0.6, *p* = 1, *p* = 0.4, and *p* = 0.6).

## Discussion

Few studies related to sensitive scalp have been published in the literature, but sensitive skin is frequently located on the scalp. The frequency of sensitive scalp without skin dermatosis was 30% in our study, as measured by the response “very sensitive” or “sensitive,” and 41.3% of the subjects had a 3S score ≥2. In the 27 patients with scalp dermatosis, 59% of the subjects had a “very sensitive” or “sensitive” scalp. In a recent study in which subjects were asked, “Do you have sensitive scalp?,” 56% had a positive answer (12). Other studies reported frequencies of 32% (11), 36% (12), or 44.2% (6), with a higher frequency in women. These frequencies were overestimated because the authors did not exclude subjects presenting with scalp dermatosis, such as psoriasis or seborrheic dermatitis. In the majority of studies, the subjects were not examined. In the study that was designed to develop the 3S score, some subjects suffered from hair loss, dandruff, seborrheic dermatitis or psoriasis. Later, a consensual definition of sensitive skin stated that “these unpleasant sensations cannot be explained by lesions attributable to any skin disease.” This definition of sensitive skin is applicable to all locations, including the scalp. In our study, the dermatological examination allowed us to include only subjects without any skin diseases on the scalp, in accordance with the new definition of sensitive skin.

We chose to include only women to focus on a homogenous population because sensitive scalp affects mostly women (9) and because they use twice as many hair cosmetic products (four products in mean) as men do (two in mean) (16).

Sensitive skin is mainly a subjective syndrome, and the diagnosis is commonly made by interviewing subjects and asking them whether they have sensitive skin. Objective tests with lactic acid or capsaicin are difficult in the presence of hairs. The sensitive scalp score (3S) was developed in 2011 as a tool to diagnose sensitive scalp, and more than 2,100 subjects were included. In the majority of studies on sensitive skin, particularly those conducted by phone or web survey, the subjects were asked whether they had very sensitive, sensitive, slightly sensitive or not sensitive scalp. The 3S has the advantage of being more precise. In our study, itching was the factor that most strongly correlated with the 3S score and was present in 90.9% of subjects, which is more frequent than on the face or other glabrous locations. Our study also showed that the scores of the subjects with sensitive scalp and those without sensitive scalp were similar. Consequently, it is not possible to define a cut-off for the diagnosis of sensitive scalp according to the 3S scores.

In our study, we did not find any risk factor for the presence of sensitive scalp. Regarding age, a study including 369 subjects in China showed that sensitive scalp were more frequent at 30–39 years of age and that the prevalence decreased after this age (17). Other studies showed that the frequency of sensitive scalp increased (14) or decreased with age (18).

We found a positive correlation between SS10 and 3S scores, suggesting that sensitive skin and a sensitive scalp are linked. The presence of sensitive scalp was positively correlated with the BoSS score, suggesting that sensitive scalp reduce quality of life. However, these results should be interpreted with caution because there are many low 3S scores and very few high 3S scores, which may impact the correlation.

In a study including 125 Korean adult women, the major triggering factor cited was hair care products, as it was cited as a triggering factor in 65.6% of subjects in the group with sensitive scalp (12). There are several types of cosmetics care products for the hair: the hair conditioners, the masks, the oils, the lotions, the serums. We have chosen to analyze the hair conditioners and masks because they are applied right down to the roots of the hair to the skin whereas the other are applied only on the hair. Because of the presence of surfactants and other potentially irritant substances, shampoos are supposed to be highly involved in the development of sensitive scalp, but this hypothesis was not supported in a previous study (14), and our exposure study did not show that shampoos are involved. However, we found a significant difference regarding amount used of hair conditioners, higher in the sensitive scalp group. These cosmetic products are used after the shampoo and are rinsed. We did not confirm the involvement of hair dyes, which we previously showed (18). An analysis of the composition of the various ingredients contained in products used for the scalp may provide more complete information on the link between sensitive scalp and cosmetics. It is not possible to conclude that there is a causal link between a cosmetic product and sensitive scalp. It is possible that hair conditioners cause the sensitive scalp, but it is also possible that subjects with a sensitive scalp use products to relieve their symptoms as hair conditioners.

Concerning consumption habits, the subjects with sensitive scalp seemed to buy organic products or those designed for children more often than those without sensitive scalp, but the difference was not significant, perhaps because of the limited sample size. There was no significant difference concerning the use of products labeled for sensitive scalp between the two groups, but there are quite a few products labeled for the sensitive scalp, contrary to sensitive skin. There was no significant difference concerning the point of purchase, whereas the subjects with non-sensitive scalp seemed to buy products more often in supermarkets. Among the limitations, there is a risk of recruitment bias. We cannot exclude the possibility that more people with sensitive skin responded to the announcement than those without sensitive skin, but we specified that all persons, with or without sensitive scalp, were invited to participate.

In conclusion, itching is the main symptom of sensitive scalp. The frequency of sensitive scalp was lower than that previously described when the subjects with scalp dermatosis were excluded. The exposure to hair conditioners is significantly higher in subjects with sensitive scalp, suggesting that sensitive scalp might be specifically related to products that are in contact with scalp for a long time.

## Data Availability Statement

The original contributions presented in the study are included in the article/supplementary material, further inquiries can be directed to the corresponding author/s.

## Ethics Statement

The studies involving human participants were reviewed and approved by ethics committee of Brest, France (29BRC18.0078). Written informed consent for participation was not required for this study in accordance with the national legislation and the institutional requirements.

## Author Contributions

A-SF, EB, LM, and A-CR contributed to the design of the research. EB and CL contributed to include subjects. EB and A-SF wrote the manuscript. All authors validated the final version of the manuscript.

## Conflict of Interest

LM received grants and consultancy fees from Bioderma, Expanscience, Galderma, Pierre Fabre, and La Roche-Posay. The remaining authors declare that the research was conducted in the absence of any commercial or financial relationships that could be construed as a potential conflict of interest.
